# About Amputations of the Leg

**Published:** 1918-12

**Authors:** 


***About Amputations of the Leg.*** By. M. PHOCAS. *Bulletin de la Société de Chirurgie de Paris.* May, 1918.

M. PHOCAS finds some advantage in placing the patient on his abdomen, instead of on his back, as is generally done for the amputations of the leg. Anesthesia, he says, has always proved very simple in that position. The leg is left hanging down naturally, and M. PHOCAS finds this more convenient than having the leg held upright by an aid, as is necessary when the patient lies on his back.

